# Synthesis and Characterization of Folic Acid-Functionalized DPLA-co-PEG Nanomicelles for the Targeted Delivery of Letrozole

**DOI:** 10.1021/acsabm.3c00041

**Published:** 2023-04-24

**Authors:** Neda Rostami, Mohammad Mahmoudi Gomari, Majid Abdouss, Alaa Moeinzadeh, Edris Choupani, Reza Davarnejad, Reza Heidari, Sidi A. Bencherif

**Affiliations:** † Department of Chemistry, Amirkabir University of Technology, Tehran 1591634311, Iran; ‡ Department of Medical Biotechnology, Faculty of Allied Medicine, Iran University of Medical Sciences, Tehran 1449614535, Iran; § Department of Tissue Engineering and Regenerative Medicine, Faculty of Advanced Technologies in Medicine, Iran University of Medical Sciences, Tehran 1449614535, Iran; ∥ Department of Chemical Engineering, Faculty of Engineering, Arak University, Arak 3848177584, Iran; ⊥ Research Center for Cancer Screening and Epidemiology, AJA University of Medical Sciences, Tehran 1411718541, Iran; # Department of Chemical Engineering, Northeastern University, Boston, Massachusetts 02115, United States; ¶ Department of Bioengineering, Northeastern University, Boston, Massachusetts 02115, United States; ∇ Harvard John A. Paulson School of Engineering and Applied Sciences, Harvard University, Cambridge, Massachusetts 02138, United States; ○ Sorbonne University, UTC CNRS UMR 7338, Biomechanics and Bioengineering (BMBI), University of Technology of Compiègne, Compiègne 60203, France

**Keywords:** nanomicelles, breast cancer, letrozole, folic acid, targeted delivery

## Abstract

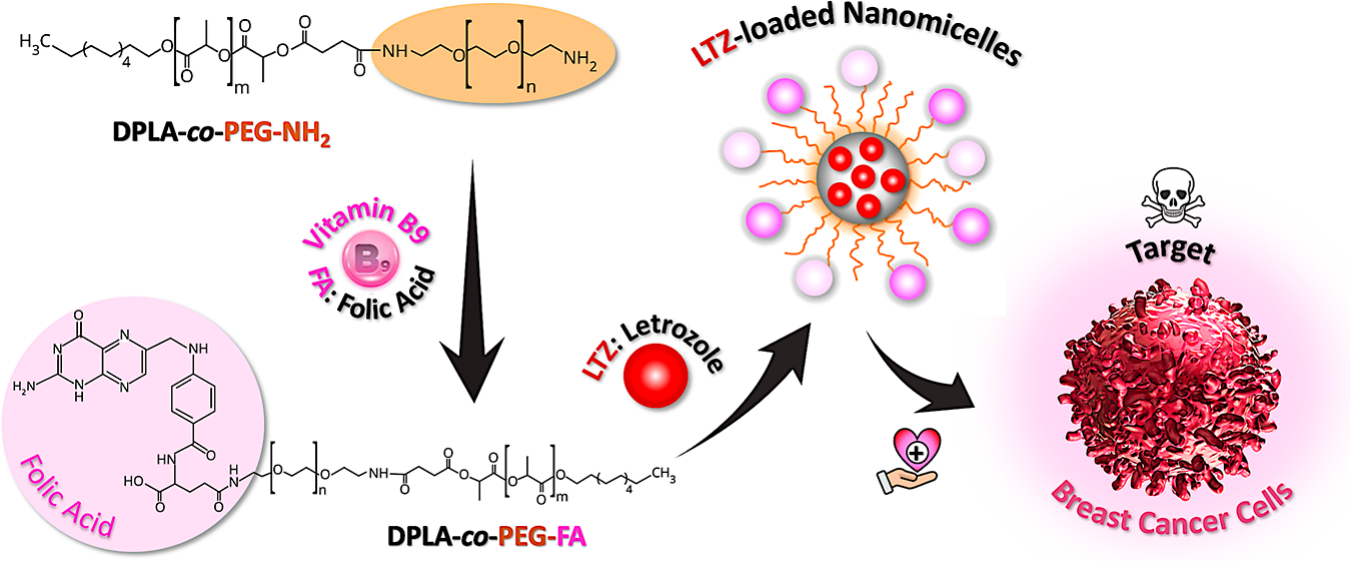

An effective treatment for hormone-dependent breast cancer is chemotherapy using cytotoxic agents such as letrozole (LTZ). However, most anticancer drugs, including LTZ, are classified as class IV biopharmaceuticals, which are associated with low water solubility, poor bioavailability, and significant toxicity. As a result, developing a targeted delivery system for LTZ is critical for overcoming these challenges and limitations. Here, biodegradable LTZ-loaded nanocarriers were synthesized by solvent emulsification evaporation using nanomicelles prepared with dodecanol-polylactic acid-co-polyethylene glycol (DPLA-co-PEG). Furthermore, cancer cell-targeting folic acid (FA) was conjugated into the nanomicelles to achieve a more effective and safer cancer treatment. During our investigation, DPLA-co-PEG and DPLA-co-PEG-FA displayed a uniform and spherical morphology. The average diameters of DPLA-co-PEG and DPLA-co-PEG-FA nanomicelles were 86.5 and 241.3 nm, respectively. Our preliminary data suggest that both nanoformulations were cytocompatible, with ≥90% cell viability across all concentrations tested. In addition, the amphiphilic nature of the nanomicelles led to high drug loading and dispersion in water, resulting in the extended release of LTZ for up to 50 h. According to the Higuchi model, nanomicelles functionalized with FA have a greater potential for the controlled delivery of LTZ into target cells. This model was confirmed experimentally, as LTZ-containing DPLA-co-PEG-FA was significantly and specifically more cytotoxic (up to 90% cell death) toward MCF-7 cells, a hormone-dependent human breast cancer cell line, when compared to free LTZ and LTZ-containing DPLA-co-PEG. Furthermore, a half-maximal inhibitory concentration (IC50) of 87 ± 1 nM was achieved when MCF-7 cells were exposed to LTZ-containing DPLA-co-PEG-FA, whereas higher doses of 125 ± 2 and 100 ± 2 nM were required for free LTZ and LTZ-containing DPLA-co-PEG, respectively. Collectively, DPLA-co-PEG-FA represents a promising nanosized drug delivery system to target controllably the delivery of drugs such as chemotherapeutics.

## 1 Introduction

Epithelial cells that line the granular ducts (85%) or lobules (15%) in breast tissues play an important role in breast cancer occurrence.^1^ In the initial stage, cancerous cell growth is restricted to the ducts or lobules, a state where cancer does not cause any symptoms and has limited potential to spread (metastasis).^2^ Eventually, cancer cells would invade the surrounding breast tissues and spread to nearby lymph nodes (regional metastasis) or other organs (distant metastasis).^3^ In this case, cancer treatment becomes challenging as various treatments are required. In fact, breast cancer treatment is generally highly effective, especially when it is detected and treated early.^2^ Standard breast cancer treatment usually involves surgical removal, radiation therapy,^4^ and medication (hormonal therapy, chemotherapy, or targeted immunotherapies).^5,6^ These therapies have the potential to inhibit tumor cell growth and prevent metastasis.^7−10^

The concept of targeted drug delivery has paved the way for innovative treatment options and techniques developed to increase therapeutic efficacy while minimizing damage to normal and healthy tissues.^11^ Drug delivery is a field of pharmaceuticals based on material-based delivery systems to enhance patient health by improving the delivery of a therapeutic to its target site, minimizing off-target accumulation, and facilitating patient compliance.^12^ Hence, the delivery strategy has a substantial effect on drug effectiveness and safety.^13^ The most significant pharmacological properties of drugs can be enhanced by using a smart drug delivery system.^14^ Over the past years, various drug delivery methods have been developed and optimized.^11^ In particular, drug delivery technologies leveraging nanoparticles have revolutionized the cancer treatment arena.^15^ Various NPs in the form of polymers,^16^ liposomes, dendrimers or carbon materials,^17^ and magnetic materials have been used as drug carriers.^18^ Nanomicelles, one of the most popular NPs, have attracted much attention due to their ability to deliver poorly water-soluble drugs controllably.^19^ Nanomicelles are composed of polymer- or lipid-based amphiphilic molecules consisting of a hydrophobic core and a hydrophilic tail.^20^ The physical and chemical properties of micelles are determinative of their function for various drugs.^21^ For instance, polyethylene (PEG), polylactic acid (PLA), poly (lactic-co-glycolic acid) (PLGA), and polyimide (PI) have been widely investigated for engineering biomaterials, including nanomicelles, in the context of targeted delivery for a variety of drugs, especially hydrophobic molecules.^22−25^

To improve cancer treatment efficacy, drug delivery nanosystems with cancer-targeted ligands can achieve more effective delivery to tumor cells.^20^ An approach consists in functionalizing NMs to selectively target key receptors in cancer cells, such as FA receptors (FRs).^26^ FA, the synthetic form of vitamin B9 (folate), is essential for cell proliferation and the biosynthesis of nucleotides.^27^ Physiologically, FA is transported into cells via FR-mediated endocytosis.^28^ FRs are typically overexpressed in human carcinomas, including ovary, kidney, lung, and breast cancer.^29^ As a result, FRs have been an attractive target for tumor-specific drug delivery.^30^

FA-conjugated nanocarriers such as PLGA-co-PEG-FA have found many applications in the field of drug delivery.^31^ PLA-co-PEG-FA, another type of FA-functionalized nanomicelles, has been designed as a nanocarrier to target folate receptors overexpressed on various cancer cells and deliver cytotoxic drugs.^32^ This approach allowed an efficient and selective delivery of doxorubicin, a standard chemotherapeutic agent used in the clinic.^33^ As such, this approach is expected to be applicable to other promising anticancer drugs such as letrozole (LTZ).^34^ LTZ is a commonly used drug for postmenopausal women with breast cancer.^35^ It lowers estrogen, thereby slowing down the growth of estrogen-positive breast tumors.^36^ LTZ is usually used after surgery by breast cancer patients for many years.^37^ However, the long-term use of LTZ is associated with several side effects.^38^ Therefore, considering the significant role of LTZ in the treatment and prevention of breast cancer recurrence,^39^ improving its function while reducing adverse side effects is critical.^40^ One potential approach is to design a drug delivery system, such as nanocarriers, to potentially reduce LTZ-associated side effects.^41^ In this work, the potency of DPLA-co-PEG and DPLA-co-PEG-FA as biodegradable micelle-based nanocarriers for the controlled delivery of LTZ against MCF-7 cells was evaluated.

## 2 Methods and Materials

### 2.1 Materials

Medical grade DPLA (14.7 kDa), 4-dimethylaminopyridine (DMAP), *N*-hydroxy-succinimide (NHS), *N*,*N*′-dicyclohexylcarbodiimide (DCC), succinic anhydride (SCA), poly(ethylene glycol) diamine (H_2_N-PEG-NH_2_, 3 kDa), 3-(4,5-dimethyl-2-thiazolyl)-2,5-diphenyl-2-H-tetrazolium bromide (MTT), polyvinyl alcohol (PVA), deuterated chloroform (CDCl_3_), and FA were purchased from MilliporeSigma. Dichloromethane (CH_2_Cl_2_), dimethylsulfoxide (DMSO), acetone, and ethanol (EtOH) were purchased from Jinan Daigang Biomaterial Co., Ltd. Human breast cancer cells (MCF-7), nontumorigenic human breast epithelial cells (MCF-10A), fetal bovine serum (FBS), penicillin, streptomycin, and trypsin were provided by the Pasteur Institute of Iran. LTZ was kindly donated by Aburaihan Pharmaceutical Co.

### 2.2 Synthesis and Characterization of DPLA-co-PEG-NH_2_ and DPLA-co-PEG-FA

#### 2.2.1 Synthesis of Carboxylic Acid-Terminated DPLA (DPLA-COOH)

First, SCA, DCC, DMAP, and hydroxyl-terminated DPLA (molar ratio = 1.1:1.1:1:1) were dissolved and reacted in CH_2_Cl_2_ (20 mL/g). The reaction proceeded for 24 h at room temperature (RT) under vigorous mixing.^42^

#### 2.2.2 Synthesis of amine-terminated DPLA-co-PEG (DPLA-co-PEG-NH_2_)

H_2_N-PEG-NH_2_, NHS, and DCC (molar ratio to the initial mole of DPLA = 1:1.1:1.1:1) were added into the previous reaction mixture of DPLA-co-PEG and allowed to react for 24 h at RT. DPLA-co-PEG-NH_2_ was precipitated three times in dH_2_O and then in CH_2_Cl_2_/EtOH (50:50), retrieved by centrifugation (8000 rpm, 1 min), and subsequently dried in a vacuum oven at RT.^42^

#### 2.2.3 Synthesis of Folic Acid-Terminated DPLA-co-PEG (DPLA-co-PEG-FA)

**Figure 1:**
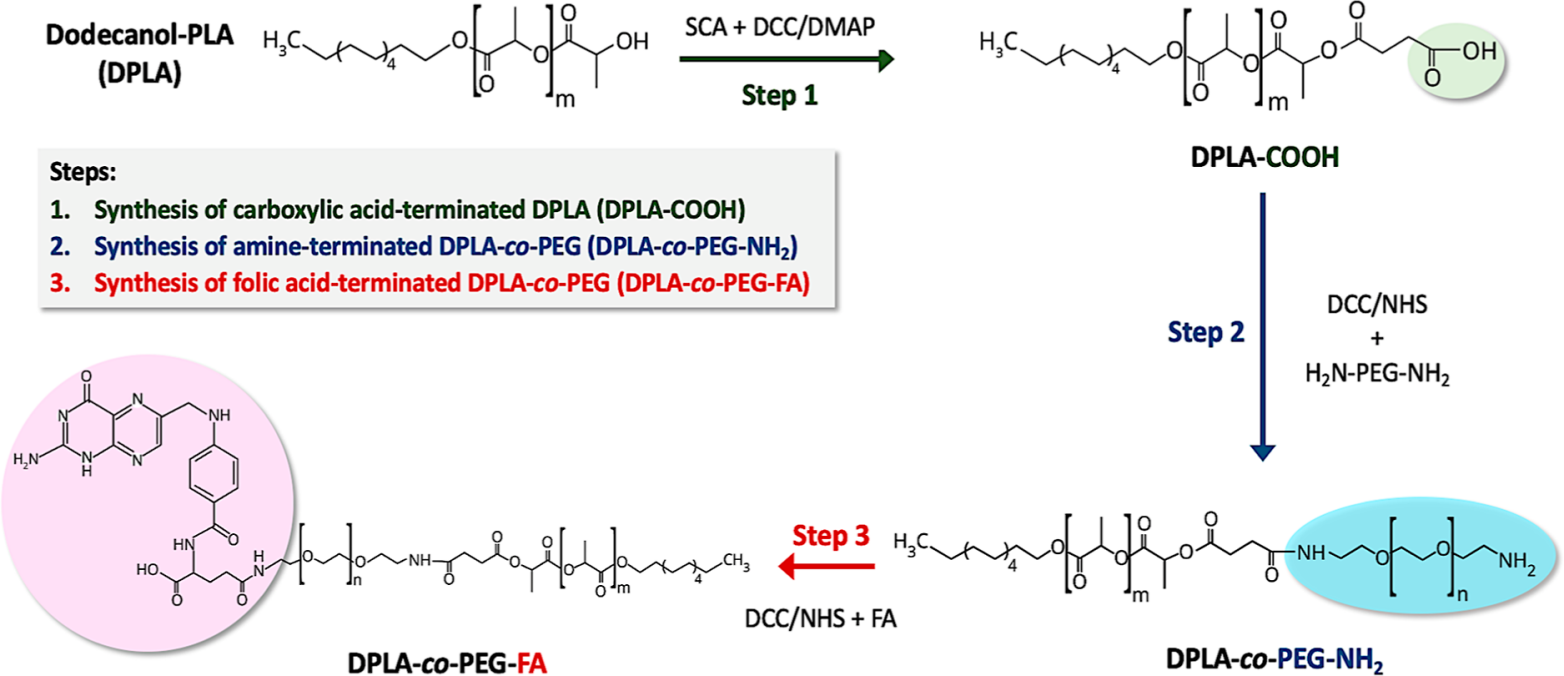
Streamlined process for the synthesis of DPLA-co-PEG-NH_2_ and DPLA-co-PEG-FA. The synthesis of DPLA-co-PEG-NH_2_ and its functionalization with FA require several chemical reactions (steps: 1, 2, and 3).

DPLA-co-PEG-NH_2_ from the previous reaction, NHS, DCC, and FA (molar ratio: 1:1.1:1.1:1) were dissolved in CH_2_Cl_2_ (20 mL/g) and allowed to react for 18 h at RT in the dark (Figure 1). Next, DPLA-co-PEG-FA was precipitated three times in dH_2_O and CH_2_Cl_2_:EtOH (50:50), retrieved by centrifugation (8000 rpm, 1 min), and finally dried in a vacuum oven at RT.^42^

### 2.3 Characterization

The synthesis and functionalization of the block copolymers were confirmed by nuclear magnetic resonance (NMR) and infrared spectroscopy (IR). IR was performed to characterize DPLA-co-PEG-NH_2_ using a Nicolet Fourier-transform IR (FTIR) spectrometer in a wavenumber range of 400 to 4000 cm^–1^ with a resolution of 4 cm^–1^. The dried sample was mixed with KBr crystals and pressed into pellets before measurements. NMR with high resolution was used to confirm the synthesis of DPLA-co-PEG-FA. ^1^H NMR spectra were recorded on a Bruker Ac Spectrometer operated at 500 MHz, and CDCl_3_ was used as a solvent.

### 2.4 Preparation and Characterization of Nanomicelles

DPLA-co-PEG-NH_2_ nanomicelles (NM) and FA-conjugated DPLA-co-PEG nanomicelles (FNM) were prepared by solvent evaporation. Briefly, 1 mL of EtOH containing NM or FNM (40% w/v) was added to 20 mL of dH_2_O and subsequently stirred for 4 h at 25 °C. While the solvent evaporated, the block copolymers self-assembled to form NM and FNM. Next, the nanomicelles were retrieved by centrifugation (8000 rpm, 3 min), washed three times in dH_2_O, and freeze-dried for 3 days. To assess their mean diameter and ζ potential, NM and FNM nanomicelles were suspended in DMSO to prevent aggregation and subsequently characterized by dynamic light scattering (DLS, Malvern Zeta sizer 3000HS, Malvern, UK). Next, their morphology was imaged by scanning electron microscopy (SEM, FEI, California, USA). Finally, contact angle measurements were performed (SDC100, Minder Hightech, China) to evaluate their hydrophobic or hydrophilic characteristics.

### 2.5 Preparation of LTZ-Containing Nanomicelles

A double emulsion method was employed to prepare LTZ-containing NM (LTZ-NM) and LTZ-containing FNM (LTZ-FNM). Briefly, 1 mL of dH_2_O and EtOH (50:50 v/v) containing 5.4 mg LTZ was added drop-wise into 5 mL of CH_2_Cl_2_ containing 54, 27, or 18 mg DPLA-co-PEG or DPLA-co-PEG-FA copolymer (DPLA-co-PEG or DPLA-co-PEG-FA/LTZ at various ratios of 10:90, 20:80, 30:70 w/w). At this stage, the solution was vortexed thoroughly for 1 min until a homogeneous mixture was obtained. Next, 2 mL of a PVA solution (3% w/v in dH_2_O) was added and vortexed again for 1 min to create a double emulsion and subsequently stirred for 30 min. To dilute the emulsion, 5 mL of a 0.2% (w/v) PVA solution in dH_2_O was added under rapid mixing for 10 min. Additionally, the organic solvents (CH_2_Cl_2_ and EtOH) were evaporated using a desiccator evaporator. Next, LTZ-loaded nanomicelles were washed three times with dH_2_O and centrifuged at 8000 rpm for 6 min. Finally, LTZ-NM and LTZ-FNM were freeze-dried for 1 day and stored at −60 °C until further use.

### 2.6 Encapsulation Efficiency

Lyophilized LTZ-NM and LTZ-FNM were first dissolved in EtOH/PBS (10:90 v/v). Next, the solutions were collected by centrifugation at 10,000 rpm for 10 min. Subsequently, UV–vis spectrophotometry (DR6000, USA) at 239 nm was used to measure the concentration of LTZ in the supernatant. The drug loading (DL) and encapsulation efficiency (EE) were calculated based on the following equations:
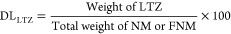
1
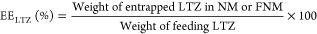
2

### 2.7 In Vitro Drug Release Kinetics

The dialysis method was used to determine the in vitro drug release kinetics of LTZ-loaded nanomicelles. Briefly, 20 mL of LTZ-loaded NM or FNM (0.5 mg/mL) was added to dialysis membranes (MWCO 12 kDa, MilliporeSigma). Next, the dialysis membranes were suspended in 30 mL PBS (pH 7.5) at 37 °C under gentle shaking (Taitec, BR-42FL, Japan). At various time points (up to 50 h), 1 mL of PBS was collected to quantify the amount of released LTZ by an ultraviolet–visible (UV–vis) spectrophotometer at 240 nm, and 1 mL of PBS was immediately added to keep the volume constant. To ensure a constant surface area during dialysis, the lengths of all dialysis membranes were kept constant. Although the drug release model was expected to follow the Higuchi model, other models, such as the zero-order and first-order Korsmeyer-Peppas models, have been evaluated to achieve a more reliable model.

### 2.8 Cell Culture

Human breast cancer cell line MCF-7 and nontumorigenic human breast epithelial cells MCF-10A were used to investigate the anticancer activity of LTZ-containing nanomicelles. Cells were cultured in DMEM medium with high glucose supplemented with 10% FBS and 1% penicillin/streptomycin at 37 °C, 5% CO_2_, and 95% humidity.

### 2.9 In Vitro Cytotoxicity

The MTT assay was used to determine the cytotoxicity of designed nanomicelles. Briefly, cells were cultured under physiological conditions until they reached 70–80% confluency. Next, MCF-7 and MCF-10A cells (7500 cells/well) were incubated with LTZ-free, LTZ-NM, or LTZ-FNM (25–175 nM) for 2 days. An MTT solution (5 mg/mL MTT in sterile PBS) was added to each well (20 μL/well) and incubated for 4 h at 37 °C. Free LTZ was used as a control in this study. After incubation, the medium was removed from each well; then, 60 μL DMSO was added and then incubated for 15 min with gentle shaking. All samples were analyzed at 590 nm using a plate reader (+MR4, Hiperion, Germany). The absorbance background values at 620 nm were subtracted from those at 590 nm for all groups. Cell viability and cytotoxicity were calculated based on the following equations:
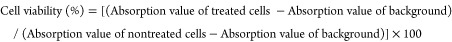
3
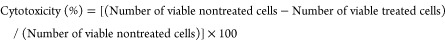
4

### 2.10 Statistical Analysis

Unless otherwise indicated, all values were expressed as mean (*n* = 3–4) ± standard deviation (SD). Statistical analyses were performed using Social Sciences software, version 21 (SPSS Inc, Chicago, IL). Significant differences between groups were analyzed by one-way analysis of variance (ANOVA) and the Student’s *t*-test. Differences were considered significant at *p* < 0.05.

## 3 Results and Discussion

### 3.1 Synthesis and Characterization

**Figure 2:**
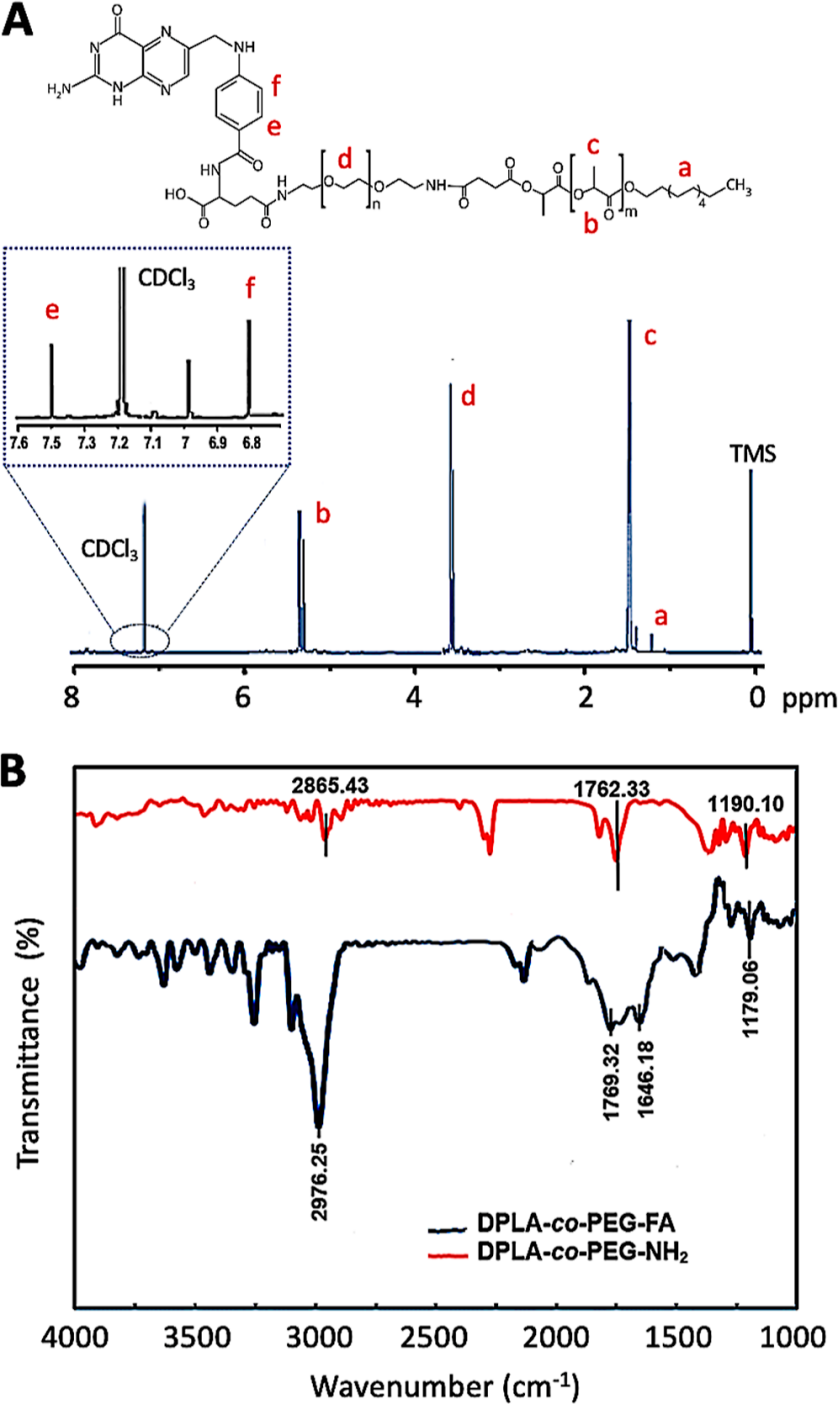
Characterization of DPLA-co-PEG and DPLA-co-PEG-FA. (A) ^1^H NMR spectrum of DPLA-co-PEG-FA in CDCl_3_. (B) Transmittance FTIR spectra of DPLA-co-PEG-NH_2_ and DPLA-co-PEG-FA.

The successful synthesis of DPLA-co-PEG-FA was confirmed by ^1^H NMR. As depicted in Figure 2, the peak around 1.35 ppm is attributed to the CH_2_ protons from dodecanol in the DPLA block. Additionally, the peaks around 1.6 and 5.2 ppm are assigned to −CH_3_ and −CH– protons in the PLA block, respectively. Lastly, the broad peak around 3.55 ppm is attributed to the repeating −O–CH_2_–CH_2_ units of the PEG, whereas the small peaks at 6.9 and 7.5 ppm are the typical aromatic protons of FA.

FTIR analysis was used to confirm the functionalization of DPLA-co-PEG with FA (Figure 2B). For both block copolymers, PLA shows characteristic stretching frequencies for ester C=O at 1762 and 1646 cm^–1^ for DPLA-co-PEG and DPLA-co-PEG-FA, respectively. Furthermore, it is worth noting that DPLA-co-PEG-FA is also characterized by an additional stretching frequency of 1769 cm^–1^, most likely due to the amide C=O stretch. Additionally, DPLA-co-PEG-FA shows a strong carboxylic acid O–H stretch at 2976 cm^–1^, which is associated with the free carboxylic acid from FA.^43^ Overall, this set of data confirms the successful formation and functionalization of the block copolymers.

### 3.2 Size and Charge Measurements

**Figure 3:**
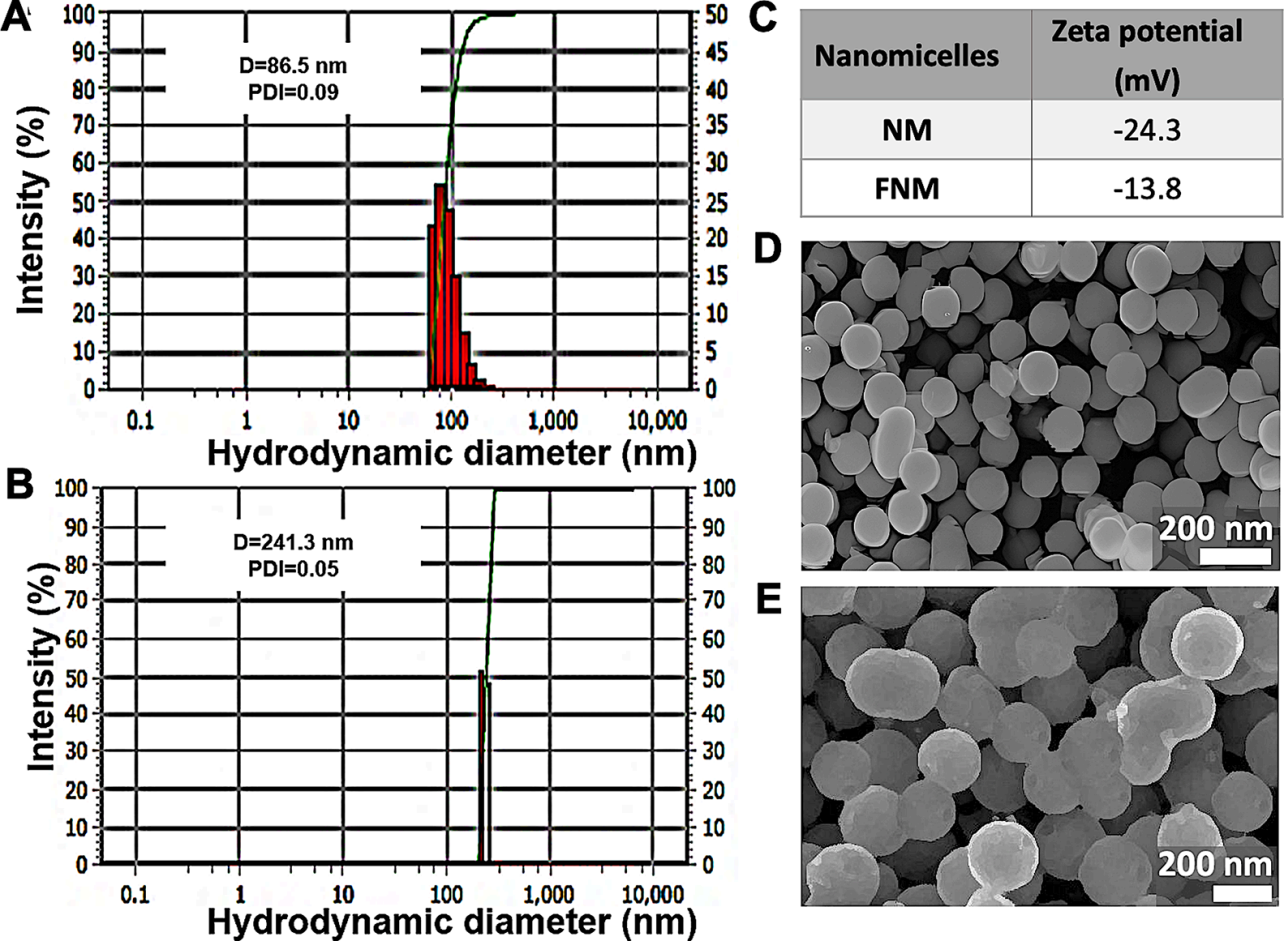
Particle size analysis and morphological observations. (A,B) Average particle size diameter and polydispersity index (PDI) of NM (A) and FNM (B). (C) ζ potential measurements of NM and FNM. (D–E) SEM images of NM (D) and FNM (E).

DLS was used to characterize the size and surface charge of DPLA-co-PEG and DPLA-co-PEG-FA nanomicelles (i.e., NM and FNM). As shown in Figure 3A,B, average particle sizes (i.e., hydrodynamic diameters) of NM and FNM are 86.5 and 241.3 nm, respectively. The topological polar surface areas of FA, PLA, and PEG have been reported to be 209, 58, and 41 Å^2^, respectively.^44−46^ This supports our finding, as the incorporation of FA into NM will result in more hydrophilic FNM and increased diameters, most likely due to higher water absorption.

Additionally, the functionalization with FA had an impact on the ζ potential of NM and FNM (Figure 3C). PLA is the core of the NM, while PEG constitutes the shell architecture, which creates a negative charge (−24.3 mV). However, in the system containing FA, due to the bipolar nature of FA, the negative charge of the nanomicelles is reduced, which was confirmed by a lower ζ potential value (−13.8 mV). Furthermore, it was described that the hydrophilic shell of nanoparticles could prevent protein adsorption to their surfaces via steric repulsion.^47^ Therefore, it is expected that these nanomicelles, particularly FNM, may evade the reticuloendothelial system and potentially accumulate at the targeted sites, such as breast tumor tissues.

### 3.3 Morphological Assessment

The morphology of the nanomicelles was investigated by SEM (Figure 3D,E). As shown in Figure 3D, the nanomicelles were spherical and exhibited a smooth surface. Figure 3E illustrates the effect of conjugating FA to the NM. Comparable to NM, FNM also displayed a spherical morphology and a homogeneous dispersion. As previously mentioned and reported by others, nanocarriers with hydrophilic properties are advantageous due to their extended circulation time in the body and ultimately improved efficacy.^48^ To this end, NM and FNM were examined for their hydrophilic–hydrophobic properties.

**Figure 4:**
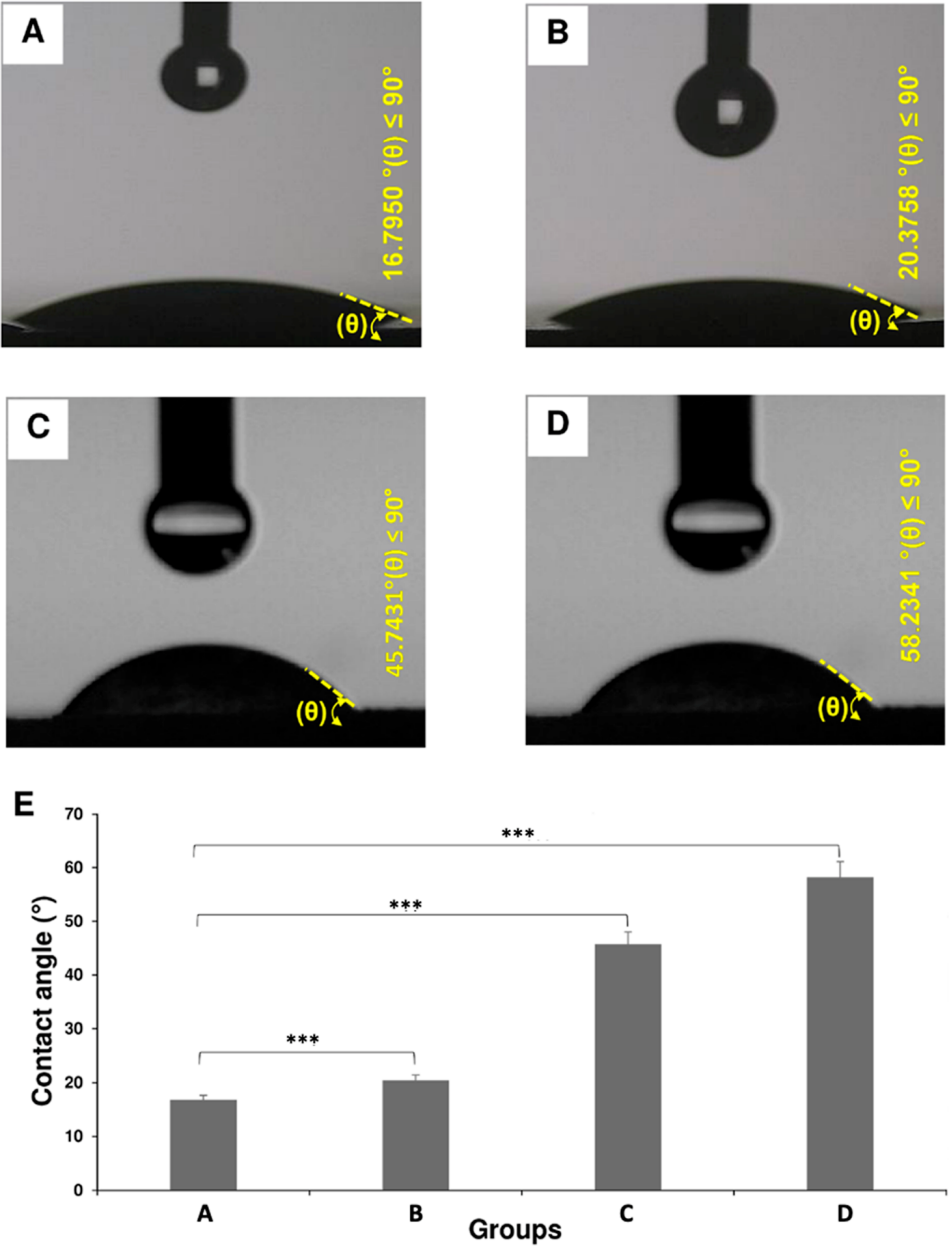
Contact angle measurements. (A–D) Contact angle images of DPLA-co-PEG (A), DPLA-co-PEG-FA (B), LTZ-containing DPLA-co-PEG (C), and LTZ-containing DPLA-co-PEG-FA (D). (E) Contact angle measurements of the LTZ-free and LTZ-loaded nanomicelles. Values represent the mean ± SD, and the data were analyzed using one-way ANOVA (*n* = 4). p*** < 0.001.

Contact angle measurements were conducted to evaluate whether the nanomicelle surfaces have a hydrophobic or hydrophilic characteristic (Figure 4). NM and FNM were both deemed hydrophilic as their contact angles were <90°. Specifically, the contact angles for NM and FNM were 16.8 and 20.4°, respectively (Figure 4A,C). When LTZ was encapsulated, the nanomicelles displayed higher contact angles (Figure 4B,D). However, nanocarriers still exhibited hydrophilic features as their contact angles remained <90°. Specifically, the contact angles for NM and FNM were 45.7 and 58.2°, respectively. This data set clearly shows the benefit of using PEG in the shell structure to increase the hydrophilic nature of the fabricated nanomicelles as drug carriers, which was not compromised even when loaded with hydrophobic LTZ.

### 3.4 Encapsulation and Loading Efficiency of LTZ

As shown in Table 1, independent of the ratio, the EE of LTZ-containing NM and FNM was found to be relatively high, ranging from 53.8 to 76.3%. It is worth noting that FNM exhibited a slightly higher EE, indicating that FA is contributing to LTZ encapsulation. The DL for LTZ-NM and LTZ-FNM at a ratio of 1:10 was found to be 4.9 and 6.9%, respectively. However, at a higher polymer content (ratio = 1:30), the LTZ loading increased to 7.9 and 10.5%, respectively. This demonstrates that LTZ can be loaded into nanomicelles and that its incorporation is proportional to the polymer concentration. This may be due to a higher viscosity of the solution and faster precipitation during the formation of the nanomicelles, leading to better LTZ entrapment. Interestingly, DL and EE values for FNM were higher when compared to NM. This may be due to the similar structures of LTZ and FA, as both contain triazole groups that may interact with each other.^49^ As a result, a higher EE was achieved for FNM.

**Table 1:** Drug Loading (DL) and Encapsulation Efficiency (EE) of LTZ at Various Drug-Polymer Ratios

Nanomicelles	Ratio (w/w) LTZ/NM or LTZ/NM	DL (%)	EE (%)
	1:30	7.9 ± 0.3	53.8 ± 2.1
**LTZ-NM**	1:20	6.4 ± 0.1	61.6 ± 3.1
	1:10	4.9 ± 0.1	69.2 ± 3.4
	1:30	10.5 ± 0.6	66.2 ± 2.9
**LTZ-FNM**	1:20	7.8 ± 0.3	70.1 ± 2.7
	1:10	6.9 ± 0.2	76.3 ± 1.4

### 3.5 Drug Release Kinetics

**Figure 5:**
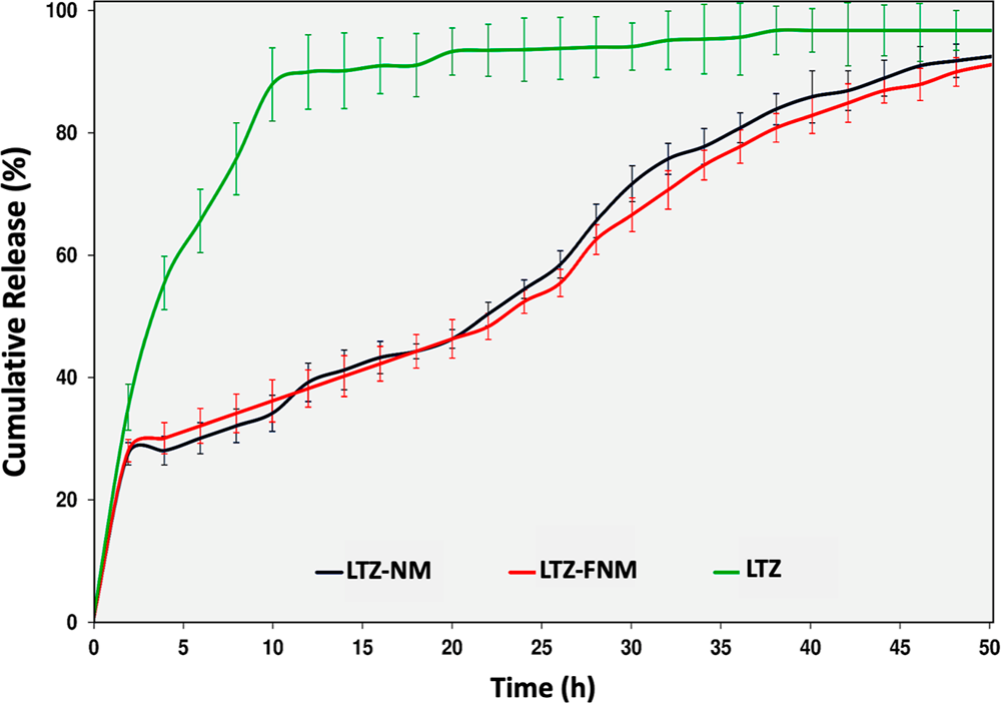
Drug release profiles. In vitro cumulative release of LTZ from LTZ-loaded nanomicelles (LTZ-NM, LTZ-FNM, and [LTZ]/[Polymer: NM or FNM] = 10:90 w/w) and free LTZ in PBS (pH 7.5) at 37 °C.

The drug release studies were conducted to determine the release profiles of LTZ from LTZ-loaded NM and FNM. As shown in Figure 5, compared to free LTZ, ∼90% of LTZ was controllably released from the nanomicelles over nearly 50 h. Approximately 30% of LTZ was rapidly released within the first few hours of the study, most likely due to a burst release. Then, the remaining payload (∼70% of LTZ) was released more sustainably. It is worth noting that both types of nanomicelles displayed similar release kinetics of LTZ, suggesting that the incorporation of FA is not significantly altering the properties of the DPLA-co-PEG copolymer.

**Figure 6:**
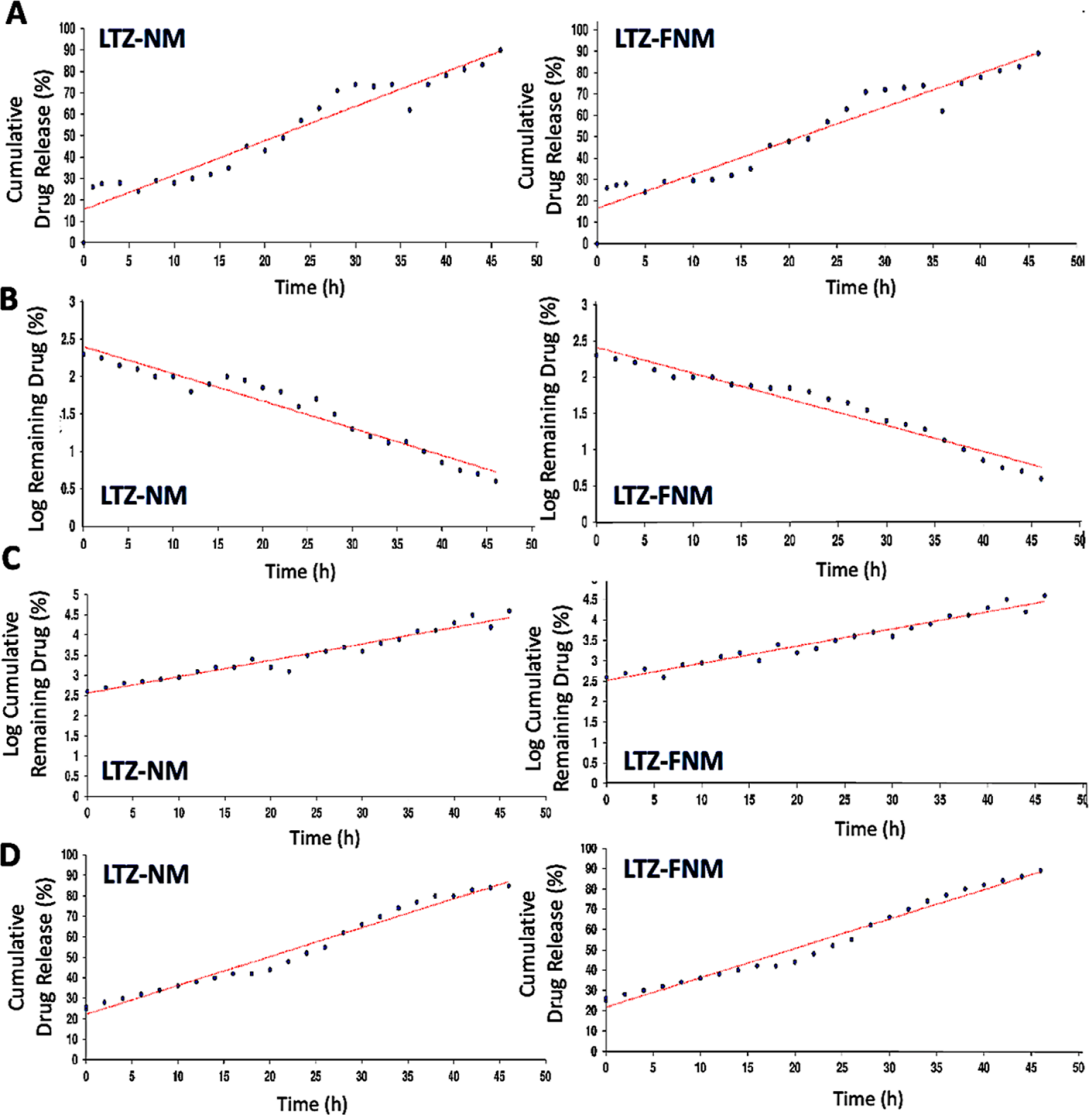
Kinetics of drug release. (A) Zero-order kinetics, (B) First-order kinetics, (C) Peppas and Korsmeyer model, and (D) Higuchi model for LTZ-NM and LTZ-FNM.

The kinetics release mechanism of LTZ from NM and FNM was assessed by fitting the in vitro release data on the mathematical equations of the zero-order, first-order, Korsmeyer-Peppas, and Higuchi (Figure 6, Table 2).

**Table 2:** Release Kinetic Models^a^

release kinetic models		LTZ-NM	LTZ-FNM
zero-order	*K*_0_	0.1005 ± 0.0031	0.0906 ± 0.0010
	*R*^2^	0.8961 ± 0.0087	0.8936 ± 0.0146
first-order	*K*_1_	0.0199 ± 0.0005	0.5447 ± 0.0638
	*R*^2^	0.5803 ± 0.0226	0.5447 ± 0.0638
Korsmeyer-Peppas	*n*^a^	0.7400 ± 0.0770	0.7700 ± 0.0770
	*R*^2^	0.9593 ± 0.0016	0.9667 ± 0.0122
Higuchi	*K*_H_	13.8720 ± 0.2220	14.560 ± 0.0593
	*R*^2^	0.9752 ± 0.0079	1.9774 ± 0.0658

a Release rate constant (*K*) and correlation coefficient (*R*^2^) values of LTZ release data obtained from various kinetic models and the “*n*” value (diffusional exponent) according to the Korsmeyer-Peppas model.

As shown in Figure 6, all in vitro release data proved a relatively good fitting on the four mathematical models. However, based on graphical representations of the cumulative percentage of drug release against time, the release of LTZ from NM and FNM correctly followed the Higuchi model, as the release profiles were very close to the trend lines.^50^ The results from Figure 6 fit well with Table 2, as the linearity (*R*^2^) values for LTZ-NM and LTZ-FNM in the Higuchi model were approximately 0.9752 and 0.9774, respectively, and were higher than the values obtained from the other models. Furthermore, the diffusion exponent “*n*” values (0.43 < *n* < 0.85) suggest that LTZ release is governed by an anomalous (non-Fickian) diffusion mechanism, in which several factors such as drug concentration could affect the drug release profile.

### 3.6 Cytotoxicity Assessment

**Figure 7:**
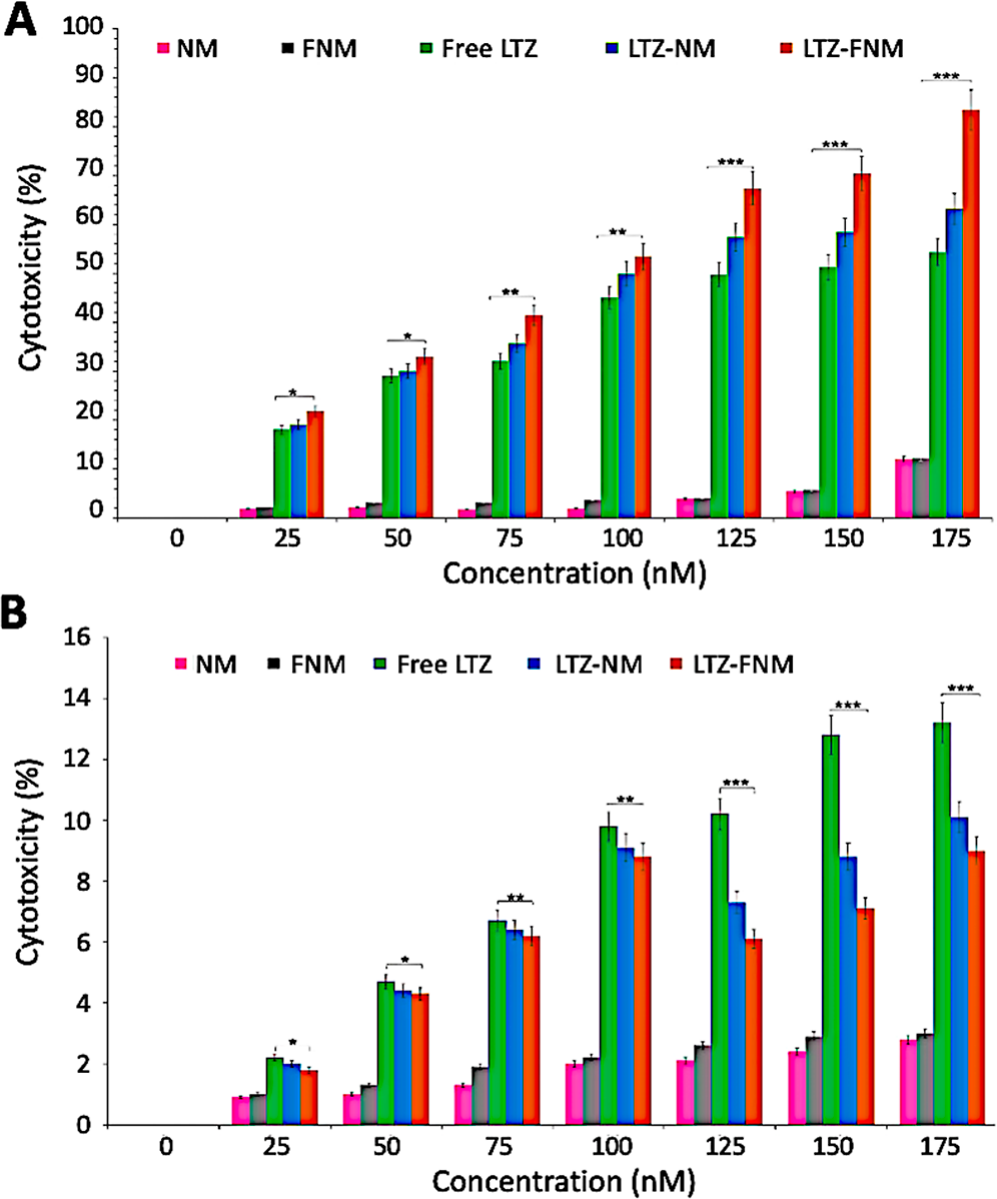
Evaluating the cytotoxic effect of LTZ-NM and LTZ-FNM on cancer cells. (A) Dose-dependent cytotoxicity of free LTZ, LTZ-NM, LTZ-FNM, NM, and FNM at various LTZ concentrations against MCF-7 cells after 48 h. (B) Dose-dependent cytotoxicity of free LTZ, LTZ-NM, LTZ-FNM, NM, and FNM at various LTZ concentrations against MCF-10A cells after 48 h. The values represent the mean ± SD, and the data were analyzed using one-way ANOVA (*n* = 3). p* < 0.05, p** < 0.01, and p*** < 0.001.

The cytotoxicity of LTZ-loaded NM and FNM was assessed against MCF-7 cells overexpressing FA receptors (Figure 7A). LTZ-NM and LTZ- FNM were tested at various LTZ concentrations (0–175 nM) and free LTZ, LTZ-free NM, and LTZ-free FNM were used as controls. LTZ-containing nanomicelles, especially FNM, outperformed free LTZ treatments and led to the highest fractions of cancer cell deaths (up to 90% at 175 nm).

The IC50 values against MCF-7 cells were found to be 87 ± 1, 100 ± 2, and 125 ± 2 nM for LTZ-FNM, LTZ-NM, and free LTZ, respectively. LTZ-FNM is likely to exhibit higher cytotoxicity against MCF-7 cells due to FR-mediated endocytosis and targeted delivery. In this process, drug uptake into MCF-7 cells is expected to increase, leading to intracellular LTZ accumulation. As expected, LTZ-NM and LTZ-FNM were found to be cytocompatible toward MCF-10A cells (Figure 7B), indicating their safety toward healthy cells but potent toxicity against cancer cells. It is worth noting that, compared to LTZ-MN and LTZ-FNM, free LTZ may be moderately uptaken more by MCF-10A cells in a nonspecific manner, most likely due to their smaller size, leading to a slight increase in cell death.

Due to tumor heterogeneity, standard monotherapies have had limited clinical success.^51^ However, combination therapy, a treatment modality that combines two or more therapeutic strategies, has been a cornerstone of cancer therapy for its additive or synergistic anticancer effects.^52^ As a result, combining several therapies with our nanomicelle-based drug delivery systems to deliver chemotherapeutics could increase treatment efficacy, prevent the development of drug resistance, and potentially reduce the duration of treatment.^53^ Therefore, more work is required to investigate further the therapeutic effect of LTZ-FNM in an animal model, examine the mode of administration (oral vs intravenous), and assess whether its efficacy could be synergized with a secondary modality, such as immunotherapy.^54^

## 4 Conclusions

In this study, we first synthesized DPLA-co-PEG and DPLA-co-PEG-FA, and then, these amphiphilic block copolymers were used to target the delivery of LTZ against cancer cells. These micelles, namely NM and FNM, exhibited a smooth and spherical morphology. The functionalization of the nanomicelles with FA resulted in slightly larger nanomicelles. These nanomicelles were used as nanocarriers for poorly water-soluble LTZ, an aromatase inhibitor used to treat hormonally positive breast cancer in postmenopausal women. LTZ was loaded into the nanomicelles with high efficiency, especially for FNM. In our release studies, approximately 90% of LTZ was gradually released over 50 h from FNM and NM. LTZ-free nanomicelles were cytocompatible toward MCF-7 and MCF-10A cells. However, when tested for their cytotoxicity with MCF-7 cells, LTZ-FNM induced higher cell deaths when compared to LTZ-NM or free LTZ. The improved antitumor activity (up to 90% cell death) is likely due to the targeted delivery of FR-targeted LTZ-FNM into the MCF-7 breast cancer cells, known to express high levels of the human FR. FA-functionalized nanomicelles represent a promising drug delivery vehicle for anticancer drugs, such as chemotherapeutics, due to their uniform size, cytocompatibility, and specificity.
